# Abdominal aortic occlusion in the setting of Covid 19 infection: literature review and a case report

**DOI:** 10.1093/jscr/rjad400

**Published:** 2023-08-08

**Authors:** Ali Kimyaghalam, Shahkar Khan, Harrison Bonilla, Kuldeep Singh

**Affiliations:** Staten Island University Hospital, Staten Island, NY, USA; Staten Island University Hospital, Staten Island, NY, USA; Staten Island University Hospital, Staten Island, NY, USA; Staten Island University Hospital, Staten Island, NY, USA

**Keywords:** infrarenal abdominal aortic occlusion, aortic thrombosis, aortic occlusion, COVID-19

## Abstract

This case report and literature review aimed to evaluate the incidence and characteristics of abdominal aortic thrombosis in patients with COVID-19. A case report was presented of a 52-y-old male with past medical history significant only for hypertension who presented with lower extremity claudication 5 months after a mild COVID-19 infection. On imaging, he had an isolated aortic thrombus and underwent successful thrombectomy. To prevent devastating limb ischemia, we emphasize early evaluation of claudication symptoms in patients with COVID-19 or recent COVID-19 infection. A literature search was performed, which yielded nine articles relevant to concomitant COVID-19 infection and abdominal aortic occlusion (AAO). The results showed that the majority of patients presented with clinical features of acute limb ischemia, along with associated features of a hypercoagulable state. While the patient age range was wide, most patients were over the age of 50 y. The case report and literature review highlight the importance of recognizing the potential for AAO in patients with COVID-19, especially in those with risk factors such as advanced age or underlying medical conditions.

## INTRODUCTION

The COVID-19 pandemic has been associated with a rise in a variety of medical complications among hospitalized patients. As is the case with many infectious states, one particularly dangerous complication is the development of an environment conducive to thrombosis. An increased incidence of arterial thrombosis in COVID-19 patients supports the development of this pro-thrombotic state. Depending on the location that is afflicted, arterial thrombosis may have different manifestations that vary in severity and symptoms. Of particular concern is the manifestation of arterial thrombosis as an abdominal aortic occlusion (AAO). AAO is a condition in which the flow of blood through the abdominal aorta becomes obstructed, leading to a profound reduction in blood supply, particularly affecting the lower extremities. Aside from the increased risk of limb loss, the mortality rate for AAO may be as high as 31% [1].

The major etiology of AAO is atherosclerosis. Atherosclerotic disease is the number one cause of vascular disease throughout the world. The pathogenesis of atherosclerosis is a complex process involving chronic stress on endothelial cells, leading to inflammation and the recruitment of macrophages. These macrophages exposed to cholesterol from oxidized low-density lipoprotein and form ‘foam cells’, which eventually contribute to the formation of an atherosclerotic plaque [2]. These plaques may subsequently rupture, leading to the exposure of pro-thrombotic material such as collagen, leading to thrombus formation and vascular occlusion. Other causes of AAO include thromboembolism, such as a large saddle embolus at the aortic bifurcation that may originate from a mural thrombus in the atrium, or traumatic injury causing a structural occlusion of the lumen of the aorta. The patient population at highest risk for AAO include those patients with the same risk factors for atherosclerosis, such as smokers, hypertensive patients, patients with dyslipidemia and males [3]. The pathophysiology of AAO involves the downstream reduction of blood flow caused by the occluding agent, whether it be a thrombus, embolus, atherosclerotic plaque or traumatic insult. The reduced blood flow can cause ischemia of the lower extremities, eventually leading to tissue necrosis, and in its most severe form, death. The ischemic insults to the lower extremities may manifest as acute limb ischemia or may develop over time as a critical limb ischemia. Patients will present with clinical features of ischemic insult, including pain in the lower extremities, along with paresthesia and weakness. Other common clinical findings include reduced or absent pulses and cool extremities [4].

The diagnostic workup of AAO includes a thorough history and physical exam, with careful attention being paid to prior surgical history, as well as the development of clinical features suggestive of acute limb ischemia. A comprehensive physical exam should be taken that includes auscultation for bruits, as well as examination of extremity pulses to detect absent or reduced pulse intensity. The primary diagnostic modality for AAO is imaging through computed tomography (CT) angiography and magnetic resonance angiography, which can properly assess the extent of the obstruction [5]. AAO is a severe condition that is frequently fatal, and management therefore includes early diagnosis with prompt surgical or endovascular interventional treatment if necessary. The goal of intervention is to remove the occluding agent and re-establish blood flow the lower extremities to prevent tissue death [6].

In this report, we present a case of a 52-y-old male who was referred to the emergency department with clinical features of AAO 5 months after a mild COVID-19 infection, along with a subsequent discussion of relevant cases in the literature highlighting potential associations between COVID-19 and an increased risk for AAO.

## CASE PRESENTATION

We present a case of a 52-y-old male with a past medical history of hypertension and hyperlipidemia, who developed lower extremity claudication and discoloration after a mild COVID-19 infection five months prior. Patient had visited an urgent care where he tested positive for COVID-19 with symptoms of a mild cough. He received no medication at the time. He was ultimately found to have significant arterial disease, with near-complete occlusion of the infrarenal abdominal aorta. This case highlights the potential vascular complications associated with COVID-19 infection and the importance of prompt diagnosis and intervention.

The patient initially reported lower extremity pain and numbness that had progressed over the course of 5 months. He experienced pain when walking about one block, which was relieved with rest. In addition, he noticed blue discoloration in his toes, initially in the fifth digit and then in the first digit bilaterally. He initially attributed this to a change in shoes, but when his symptoms worsened, he contacted his primary care physician and was referred to a vascular specialist. As his symptoms continued to progress, he presented to the emergency department (ED).

**Figure 1:**
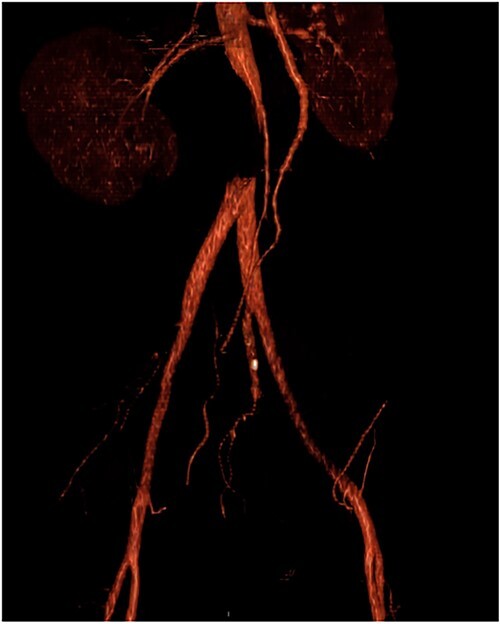
CTA with occlusion of the infrarenal abdominal aorta above the bifurcation of the iliac arteries.

**Figure 2:**
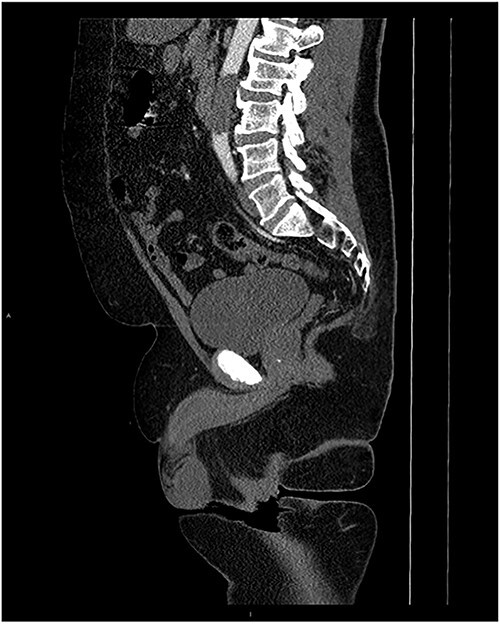
MRI Sagittal with contrast with occlusion of the abdominal aorta.

Upon admission to the ED, vital signs were stable, and an arterial duplex revealed diminished flow bilaterally in the common femoral arteries, superficial femoral artery and popliteal arteries, suspicious for aortoiliac disease. A computed tomography angiogram (CTA) with runoff was then conducted, which showed a near-complete occlusion of the infrarenal abdominal aorta below the level of the inferior mesenteric artery (Figs 1 and 2). The patient was promptly seen by vascular surgery and planned for an open aortic thrombectomy.

**Figure 3:**
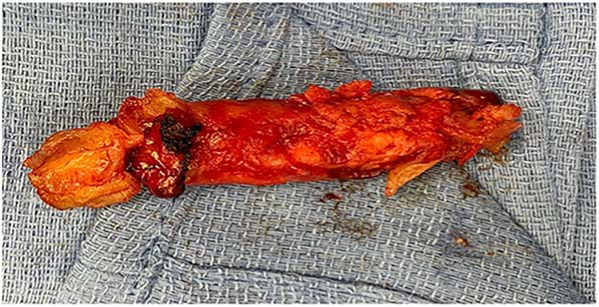
Extracted abdominal aortic thrombus.

The patient underwent an open aortic thrombectomy with a total OR time of 3 hours and 34 minutes. Estimated blood loss was 800 cc, and intravenous fluids and blood products were administered as needed. Surgical findings were significant for an aortic thrombus (Fig. 3). There were no intraoperative complications, and the patient remained hemodynamically stable throughout the procedure. Patient was discharged on post-operative day 6, with a scheduled follow-up with the vascular surgery clinic and has been back to the normal state post operatively.

## DISCUSSION

The literature suggests an association between COVID-19 and vascular complications. The pathophysiology underlying the higher risk for vascular complications in COVID-19 patients is still poorly understood. However, proposed mechanisms include a combination of endothelial injury, inflammation, and a prothrombotic state. Our patient’s history was significant only for hypertension, and his presentation of lower extremity claudication and discoloration was notable given his recent COVID-19 infection five months prior. A prompt diagnosis with surgical intervention was crucial in preventing further complications, including limb loss and death. The case highlights the importance for healthcare providers to maintain a high degree of suspicion for arterial thrombosis in COVID-19 patients with atherosclerosis, and associated risk factors such as hypertension [7].

A literature review was performed using the Pubmed database to find articles associated with the key words, ‘aorta,’ ‘aortic,’ ‘thrombus’ and ‘Covid-19.’ Within these articles, the search criteria was narrowed to articles that related to AAO in the setting of Covid-19 infection. A total of nine articles were analyzed that reported cases of abdominal aortic thrombosis in COVID-19 patients. In all, 67% of patients in the literature presented with characteristic features of acute limb ischemia. Our analysis revealed that 67% of the patients in our literature review presented with signs and symptoms of acute limb ischemia. Additionally, 67% of the patients with reported sex were male, and ages ranged from neonate to 85-y old. However, 81% of the patients were over the age of 50 (Table 1).

**Table 1:** Literature review of isolated abdominal aortic thrombosis in the setting of COVID-19 infection

	Age-Gender	Presenting Symptoms	Area Occluded	Intervention-Outcome
Article 1: Acute Aortic Thrombosis in COVID- 19	63F, 69 M, 85F	LE pain and loss of pulses, LE pain, palpable pulses, and loss of sensation; gluteal pain radiating to both lower limbs and numbness	Infrarenal abdominal aorta	Surgical procedure: extra-anatomic axillobifemoral bypass; surgical thrombectomy with Fogarty balloon through bilateral femoral access; Same as prior. Outcome: Bilateral pedal pulse recovery.
Article 2: COVID-19- Related Abdominal Aortic Thrombosis	54 M	Abdominal pain and mild nausea	Partial thrombus in 2-cm segment of abdominal aorta at supra-celiac level and splenic parenchymal infarction	Enoxaparin was started at 8000 IU/12 hour. Outcome: The thrombus completely disappeared without any additional complications in the abdominal CT carried out one month later
Article 3: Concomitant Acute Aortic Thrombosis and Pulmonary Embolism Complicating COVID- 19 Pneumonia	71 M	Dyspnea, fever, and cough of 2 weeks. On hospital day 3, developed increasing hypoxia with elevated D-dimer	Acute pulmonary embolism in the right lower lobe. Intra-aortic free-floating thrombus at the thoraco-abdominal junction	Enoxaparin. Outcome: Favorable
Article 4: Aortic Thrombosis in a Neonate with COVID- 19-Related Fetal Inflammatory Response Syndrome Requiring Amputation of the Leg: A Case Report	Neonate	Healthy full-term newborn discharged from hospital on Day 3 developed irritability and progressive blackish discoloration of the toes of the right lower limb on Day 6 of life	Doppler imaging revealed acute thrombosis of the abdominal aorta with a critically ischemic right lower limb.	The neonate was treated with corticosteroids, heparin infusion and recombinant tissue plasminogen activator, and required surgical embolectomy followed by right limb amputation. Outcome: By Day 31 of life, inflammatory markers showed serial return to normal and the neonate was discharged on oral steroids and aspirin
Article 5: Acute Thrombosis of an Aortic Prosthetic Graft in a Patient with severe COVID-19-Related Pneumonia	67 M	Bilateral lower limb ischemia	A duplex ultrasound demonstrated the complete thrombosis of the aortic graft without flow at the femoral level	An urgent angio-computed tomography scan for revascularization purpose was requested, but the patient died on the arrival in the radiological suite. Outcome: Cardiac arrest prior to surgery
Article 6: Systemic Arterial Thrombosis and Acute Mesenteric Ischemia in a Patient with COVID-19	56 unspecified	R MCA stroke; The next day the patient developed abdominal pain and vomiting	Free-floating thrombus of the aortic arch associated with occlusion of the SMA	The patient underwent endovascular thrombectomy and a laparotomy with resection of 2 mof the small bowel
Article 7: Extensive Aortic Thrombosis in a Patient with COVID-19	83F	In the nursing home, blue and pale lower extremities were noted by a physician	CT showed severe aortic thrombosis extending from the lower thoracic aorta just above the diaphragm to the abdominal aorta and the iliac arteries	In the ICU, the patient required increasing doses of vasopressors. Based on the patient’s goals of care, comfort measures were instituted. Abdominal aorta and the Outcome: Died
Article 8: Aortic Thrombosis in COVID-18	63 M	Cold foot with an absent dorsalis pedis pulse 7 days after admission for COVID-19	CTA demonstrated a large thrombus in the lower thoracic aorta (not present on CT pulmonary angiogram the preceding week)	Therapeutic dose of low molecular weight heparin (LMWH) for 6 weeks.

One of the most common presenting symptoms in the reviewed cases was acute limb ischemia, which is characterized by severe pain and loss of pulses in the affected limb. The literature describes the clinical features of acute limb ischemia with the characteristic ‘6 P’s’ of pain, pallor, pulselessness, paralysis, paresthesia and poikilothermia. For example, in one case report, a 63-y-old female was diagnosed with acute limb ischemia and loss of pulses in both lower extremities [8]. Similarly, a 67-y-old male was diagnosed with bilateral lower limb ischemia [9]. Acute limb ischemia requires urgent medical intervention to prevent tissue necrosis and limb loss, and therefore, COVID-19 patients at risk for AAOs should be closely monitored for the development of characteristic clinical features of acute limb ischemia, as timing is crucial for management [10,11].

Certain non-specific symptoms were also reported in the literature. In one case, a 54-y-old male was diagnosed with abdominal pain and mild nausea [12]. Another case report described a 71-y-old male who was diagnosed with dyspnea, fever and cough of 2 weeks, and on hospital day 3 developed increasing hypoxia with elevated D-dimer [13]. These symptoms and laboratory findings are present in many different pathologies, and therefore may indicate systemic involvement and require further evaluation to identify other underlying mechanism of disease that may be present. Interestingly, the above two cases each include patients with concomitant thrombotic diseases, with the 54-y-old patient diagnosed with associated findings of a supra-celiac abdominal aortic thrombus, as well as a parenchymal splenic infarct [12] and the 71-y-old patient developing acute pulmonary embolism in the right lower lobe and intra-aortic free-floating thrombus at the thoraco-abdominal junction [13]. The finding of concomitant thromboembolic diseases further highlights the potential risk for COVID-19 to create pathological pro-thrombotic states, further increasing the risk of disease in patients with underlying risk factors for AAO [14,15].

Although the literature review showed a wide range of ages affected by aortic occlusive disease, with some cases reported in neonates and others in elderly individuals, the majority of cases occurred in patients over the age of 50, underlying the importance of advanced age as a risk factor for aortic occlusive disease in COVID-19 patients. Clinicians should therefore maintain a particularly high degree of suspicion in elderly, male patients with concomitant COVID-19 and other risk factors for AAO.

## CONCLUSION

The presented case report highlights the potential consequences of COVID-19 infection in a patient with underlying risk factors for AAO, such as hypertension and hyperlipidemia. The literature review conducted provides similar examples of pro-thrombotic states with subsequent acute disease in patients with a history of COVID-19 infection. This study suggests the need for closer monitoring of signs of aortic thrombosis in patients with COVID-19, especially those with underlying risk factors or medical conditions, as well as the need for continued research into aortic and arterial thrombosis in the setting of COVID-19 infection.

Patient has given full consent for publication purposes.
